# Lymphoepithelioma-like, a variant of urothelial carcinoma of the urinary bladder: a case report and systematic review for optimal treatment modality for disease-free survival

**DOI:** 10.1186/s12894-017-0224-4

**Published:** 2017-04-27

**Authors:** Andy W. Yang, Aydin Pooli, Subodh M. Lele, Ina W. Kim, Judson D. Davies, Chad A. LaGrange

**Affiliations:** Division of Urologic Surgery, University of Nebraska Medical Center, Omaha, NE USA

**Keywords:** Lymphoepithelioma-like carcinoma, Bladder tumor, Systematic review, Case report

## Abstract

**Background:**

Lymphoepithelioma-like carcinoma (LELC) is a rare high-grade carcinoma that resembles nasopharyngeal lymphoepithelioma and can occur throughout the body. First reported in 1991, bladder LELC has an incidence of about 1% of all bladder carcinomas. Due to its rare occurrence, prognoses and ideal treatment guidelines have not been clearly defined.

**Methods:**

A PubMed search was performed using two terms, “lymphoepithelioma-like carcinoma” and “bladder.” Review articles, articles in foreign languages, expression studies, and studies not performed in the bladder were excluded. We report a case of LELC of the bladder including treatment and outcome and performed a systematic review of all 36 available English literatures from 1991 to 2016 including the present case to identify factors affecting disease-free survival.

**Results:**

One hundred forty cases of bladder LELC were analyzed. The mean age of the patients was 70.1 years ranging from 43 to 90 years with 72% males and 28% females. Pure LELC occurs most often at 46% followed by mixed LELC 28% and predominant LELC 26%. EBV testing was negative in all cases tested. Mean follow-up length for all cases was 33.8 months with no evidence of disease in 62.2%, while 11.1% died of disease, 10.4% alive with metastasis, and 8.2% died without disease. 5.0% of cases had recurrence at an average of 31.3 months. Prognosis is significantly favorable for patients presenting with pure or predominant forms of LELC compared to mixed type (*p* < 0.0001). The treatment significantly associated with the highest disease mortality and lowest disease-free survival was TURBT alone when compared to any multi-modality treatment (*p* < 0.01).

**Conclusion:**

We conclude that the best treatment modality associated with the highest disease-free survival is multi-modal treatment including radical cystectomy.

## Background

Lymphoepithelioma-like carcinoma (LELC) is a rare high-grade carcinoma that resembles nasopharyngeal lymphoepithelioma and has been reported to occur in other sites of the body such as gastrointestinal tract [1], liver [2], lung [3], skin [4], uterus [5], gallbladder [6], pancreas [7], kidney [8], and breast [9]. First reported in 1991 [10], LELC of the bladder appears to resemble LELC histologically in the nasopharynx but is actually a variant of urothelial carcinoma and has an incidence of about 1% of all bladder carcinomas [11]. Unlike other sites of the body, LELC in the bladder has not been associated with the presence of Epstein-Barr Virus to date [12]. Due to its rare occurrence, prognoses and ideal treatment guidelines have not been clearly defined. We report a case of LELC in the bladder and performed a systematic review of all available English literature including the present case to evaluate factors affecting disease-free survival.

## Methods

A PubMed search was performed using two terms, “lymphoepithelioma-like carcinoma” and “bladder.” Of the 63 results generated as of July 18th, 2016, 27 review articles, articles in foreign languages, expression studies, and studies not performed in the urinary bladder were excluded. Attempts were made to translate foreign articles to minimize bias but it was unsuccessful. Potential bias due to language barrier should be minimal. A total of 140 patients, including the present case, were collected from 36 published English articles from 1991 to 2016 [10, 11, 13–46]. Preferred reporting items for systematic review and meta-analysis protocols (PRISMA-P) 2015 guidelines were followed including creation of a protocol available upon request [47]. Patient data collected include gender, age, chief complaint, LELC type, TNM staging, EBV status, primary treatment, secondary treatment, neoadjuvant therapy used, follow-up time in months, recurrence time in months, and outcome. Studies with insufficient information for particular data were excluded from that particular statistical analysis to reduce bias. LELC classification criteria was described by Amin et al. with pure being 100% of the tumor showed LELC pattern, pre-dominant being ≥ 50% mixed with another type of tumor pattern, and mixed being < 50% mixed with another type of tumor pattern. *Student’s t-test* was performed for statistical analysis.

### Case report

A 69-year-old African American female presented in February 2015 in our department with the chief complaint of gross hematuria and dysuria that started in December 2014. Prior to urology evaluation, she had received two courses of antibiotics without resolution for her presenting symptoms. The patient denied history of urologic trauma, nephrolithiasis, chronic Foley catheter, family history of genitourinary (GU) malignancy, or previous GU surgeries. The patient had a history of stage IA adenocarcinoma of the right upper lung in 2011 and a 20-pack year history of smoking.

**Fig. 1:**
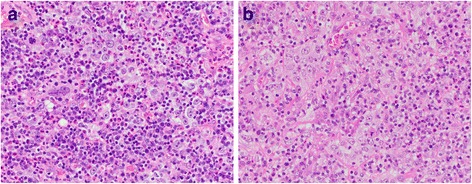
Note the (**a**) high-grade carcinoma cells with large nuclei, irregular nuclear borders and prominent nucleoli present in small aggregates and also (**b**) singly with ill-defined cytoplasmic borders imparting a syncytium-like pattern and admixed with numerous inflammatory cells, typical of a lymphoepithelioma-like urothelial carcinoma. Original magnification X400; hematoxylin and eosin stain

Cystoscopy revealed a large complex bladder mass on the right lateral wall and right trigone involving the right ureteral orifice. Abdominal and pelvic CT scan revealed right-sided bladder mass involving the right ureterovesical junction, right hydronephrosis and right-sided pelvic lymphadenopathy. Transurethral resection of the bladder tumor (TURBT) was performed and pathologic examination showed a prominent inflammatory background with admixed high-grade undifferentiated tumor cells arranged in sheets with ill-defined cytoplasmic borders imparting a syncytial appearance diagnostic of the LEL variant of urothelial carcinoma (Fig 1a and b). Foci of urothelial carcinoma in situ were also noted involving the surface urothelium. Muscularis propria invasion was present.

Patient was treated with four cycles of neoadjuvant chemotherapy of gemcitabine and cisplatin. Repeat CT two weeks after the last round of chemotherapy revealed smaller right-sided bladder mass (6.1 x 2.9 cm vs. 6.9 x 3.4 cm). Two months after the last round of chemotherapy, the patient underwent a radical cystectomy with ileal conduit diversion and pelvic lymph node dissection in July 2015. The operation was complicated by extensive adhesions from previous appendectomy, hysterectomy, and hernia repair with ventral mesh placement necessitating small bowel resection.

Final pathology report showed three high-grade tumor foci in the bladder with the largest being high-grade urothelial carcinoma located in the right lateral wall measuring 2.8 cm, another tumor located in the posterior wall measuring 0.9 cm with areas of squamous differentiation, and the smallest tumor located at the dome measuring 0.6 cm with pathology consistent with LEL urothelial carcinoma. The LEL variant of urothelial carcinoma is rare and diagnosed by the presence of high-grade/poorly differentiated tumor cells admixed with a prominent inflammatory cell infiltrate. The tumor cells have high nuclear:cytoplasmic ratios and indistinct cytoplasmic borders imparting a syncytium-like appearance. The overall appearance is similar to the lymphoepitheliomas typically seen in the nasopharyngeal region. They can be seen in the bladder either in the pure form or admixed with more usual forms of high-grade urothelial carcinoma, as seen in the present case. One obturator lymph node was positive for metastatic urothelial carcinoma (1/17 nodes positive). All surgical margins were negative. Final pathology staging was pT3bN1MX.

Patient had an uneventful recovery and was discharged on post-operative day 12 to a skilled nursing facility. Repeat abdominal and pelvis CT at 9 weeks post-op showed no mass, lymphadenopathy, or destructive osseous lesions. Patient reported improvement in appetite and normal bowel movement with persistent mild abdominal pain. However, lung cancer follow-up chest CT in September 2015 revealed a new 3 mm left upper lobe nodule not present in preoperative chest CT in June 2015. Repeat chest CT in February 2016 showed left upper lobe nodule enlarged to 12 mm. CT-guided needle biopsy showed CK7+, p40+, and GATA3+ tumor cells similar to morphology of previous bladder cancer, consistent with metastatic urothelial carcinoma. Multiple new liver lesions were present on repeat CT in April 2016. Patient unfortunately died with metastases in October 2016.

## Results

One hundred forty cases of LELC in the bladder including the present case were reported between 1991 and 2016. The mean age of the patients was 70.1 years ranging from 43 to 90 years with 57% males, 22% females, and 21% unknown; of those with known genders, 72% were male and 28% female (Table 1). Primary presentation was gross hematuria in 53% of patients. Mean follow-up length for all cases was 33.8 months with no evidence of disease in 62.2%, while 11.1% died of disease, 10.4% alive with metastasis, 8.2% died without disease, and 8.2% lost to follow-up. 5.0% of cases had recurrence at an average of 31.3 months. Pure LELC occurs most often at 46% (62 cases) followed by mixed LELC 28% (38 cases) and predominant LELC 26% (36 cases). Pathological staging of the tumor was pT1 in 10.1% (14 cases), pT2 in 56.1% (78 cases), pT3 in 30.9% (43 cases), and pT4 in 2.9% (4 cases). Lymph node metastasis was present in 13.6% of patients, with distant metastasis noted in 5.7%. EBV testing was performed in 51.4% of the cases and was negative in all cases.

**Table 1:** LELC cases from 36 published English literature from 1991–2016 including the present case with demographic breakdown

Reference	Case(s)	Reference	Case(s)
Zukerberg et al., 1991 [10]	1	Guresci et al., 2009 [22]	1
Young et al., 1991 [44]	1	Singh et al., 2009 [35]	1
Dinney et al., 1993 [19]	3	Trabelsi et al., 2009 [39]	1
Amin et al., 1994 [13]	11	Yun et al., 2010 [45]	1
Bianchini et al., 1996 [14]	1	Kozyrakis et al., 2011 [26]	6
Holmang et al., 1998 [23]	9	Williamson et al., 2011 [41]	33
Constantinides et al., 2001 [18]	3	Pantelides et al., 2012 [32]	1
Lopez-B et al., 2001 [11]	13	Mori et al., 2013 [30]	1
Ward et al., 2002 [40]	1	Spinelli et al., 2013 [36]	1
Porcaro et al., 2003 [33]	1	Yoshino et al., 2014 [43]	1
Chen et al., 2003 [16]	2	Ziouziou et al., 2014 [46]	1
Izuquierdo et al., 2004 [24]	3	Kushida et al., 2015 [27]	1
Guresci et al., 2005 [21]	1	Kessler et al., 2015 [25]	1
Yaqoob et al., 2005 [42]	1	Mina et al., 2015 [29]	1
Mayer et al., 2007 [28]	1	Raphael et al., 2015 [34]	1
Tamas et al., 2007 [38]	29	Nagai et al., 2016 [31]	1
Cai et al., 2008 [15]	2	Stamatiou et al., 2016 [37]	1
Chikwava et al., 2008 [17]	1	Yang et al., 2017	1
Fadare et al., 2009 [20]	1	Total	140
Age Range	43–90	Male	72%
Average Age	70.1	Female	28%

Comparing treatment modality, 50% of the cases utilized one treatment modality only with radical cystectomy being the most common (58.6%) followed by TURBT (30.0%), partial cystectomy (7.1%), and intravesical chemotherapy (1.4%) with outcomes of no evidence of disease (55.7%), died of disease (12.9%), alive with metastasis (10.0%), and died without disease (7.1%). Of the multi-modality treatments, primary treatments were diverse and included TURBT (49.9%), radical cystectomy (41.0%), partial cystectomy (6.0%), intravesical chemotherapy (1.5%), chemotherapy (0.8%), and radiation therapy (0.8%). Secondary treatments included chemotherapy (51.8%), radiation therapy (28.2%), TURBT (7.1%), intravesical chemotherapy (7.1%), radical cystectomy (4.7%), and thermal ablation (1.2%). Outcomes for those receiving multi-modal treatments include no evidence of disease (67.2%), alive with metastasis (10.5%), died without disease (9.0%), and died of disease (7.5%) (Table 2).

**Table 2:** Outcomes of all cases comparing single vs. multi-modal treatment modality

Treatments	NED	AWM	DOD	DWD
Single	55.7%	10.0%	12.9%	7.1%
Multi	67.2%	10.5%	7.5%	9.0%
Overall	62.2%	10.4%	11.1%	8.2%

*NED* no evidence of disease, *AWM* alive with metastasis, *DOD* died of disease, *DWD* died without disease

Comparing surgical resection methods, TURBT alone has the lowest disease-free survival rate (33.3%) when compared to any combination therapy (67.9%) (*p* < 0.01); TURBT alone also carries the highest mortality rate (23.8%) when compared to any combination therapy (7.1%, *p* < 0.05). Radical cystectomy is associated with the highest disease-free survival rate at 67.8% and is significant when compared to TURBT alone at 33.3% (*p* < 0.01) but not significant when compared to partial cystectomy at 50%.

Systematic chemotherapy treatments utilized are varied with 24 cases documenting detailed regiments. Of those, eight cases specified MVAC treatments while 16 cases specified GC treatments. Neoadjuvant chemotherapy was administered in 4.3% of cases with an average disease-free survival of 41 months and did not significantly impact outcome. Comparing subtypes of LELC, the treatment regiments reported did not significantly differ; of those with pure and predominant LELC, 71.0% and 75.0% had no evidence of disease, respectively, while only 31.6% of mixed LELC patients had the same outcome (*p* < 0.0001, Table 3). In addition, patients who underwent radical cystectomy had the highest disease-free survival (67.8%, *p* < 0.01) when compared to partial cystectomies (50%) or TURBT only (33.3%). However, patients receiving TURBT combined with any type of secondary treatment have a 71.1% disease-free survival rate.

**Table 3:** Outcomes for LELC types and treatment modalities

LELC Type	Cases	NED	*p-*value	DOD	*p-*value
Pure	62	71.0%	0.00002	1.6%	0.0001
Predominant	36	75.0%	0.00002	5.6%	0.002
Mixed	38	31.6%	-	28.9%	-
Treatment	Cases	NED	*p-*value	DOD	*p-*value
MM	112	67.9%	0.002	7.1%	0.04
RC+	59	67.8%	0.002	10.2%	0.08
PC+	8	50.0%	0.21	12.5%	0.22
TURBT+	45	71.1%	0.002	2.2%	0.012
TURBT−	21	33.3%	-	23.8%	-

*p*-values calculated against mixed type and against TURBT-only

*NED* no evidence of disease, *DOD* died of disease, *MM* multi-modality overall treatments including RC+, PC+, and TURBT+; RC+, radical cystectomy + adjuvant therapy, *PC+* partial cystectomy + adjuvant therapy, *TURBT+* transurethral resection of the bladder + adjuvant therapy, *TURBT-* transurethral resection of the bladder only

## Discussion

LELC of the bladder is a rare cancer that most often presents with painless hematuria occurring in older males. By the time of presentation, most LELCs have invaded the muscularis propia but have not metastasized outside of the bladder. Even though LELC in other organ systems has been shown to be associated with EBV, no case of LELC in the bladder has been associated with the presence of EBV. The subtypes of LELC appear to significantly impact outcome, as disease-free survival is higher in predominant and pure LELC than mixed LELC. Highest mortality is mixed LELC followed by predominant and pure LELC and this could suggest that LELC itself is not as aggressive as high-grade urothelial carcinoma.

As for treatment impacting outcome, TURBT alone should not be recommended, as it is associated with both lowest disease-free survival and highest mortality rate. Radical cystectomy is associated with the highest disease-free survival rate, whereas partial cystectomy was only utilized as the main surgical resection method in eight cases its impact is unknown. As for neoadjuvant chemotherapy, it was administered in six cases and while it had a longer disease survival, it was not significant, perhaps also due to the small sample size. As for chemotherapy regiment, it appears to have evolved over time and without significant difference as all eight cases of MVAC were before 2003 and all 16 cases of GC were after.

To define best treatment strategy for rare diseases is difficult as rare diseases are best evaluated in a prospective registry. Even though LELC in the bladder is rare and there is currently no clear treatment guideline, our study suggests that a combination therapy including radical cystectomy would possibly yield the best outcome.

## Conclusion

LELC of the bladder is a rare cancer that most often occurs in older males. Of the three subtypes, mixed LELC carries the highest mortality rate and TURBT alone or any single treatment is not recommended for therapy as it is associated with both the highest mortality rate and the lowest disease-free survival rate. Prognosis is favorable for patients presenting with pure or predominant forms of LELC and those undergoing combination therapies that include radical cystectomy while the impact of neoadjuvant chemotherapy is yet undetermined.

## Abbreviations

GU: Genitourinary
LELC: Lymphoepithelioma-like carcinoma
TURBT: Trans-urethral resection of bladder tumor.
